# DUBbing Cancer: Deubiquitylating Enzymes Involved in Epigenetics, DNA Damage and the Cell Cycle As Therapeutic Targets

**DOI:** 10.3389/fgene.2016.00133

**Published:** 2016-07-28

**Authors:** Adan Pinto-Fernandez, Benedikt M. Kessler

**Affiliations:** Target Discovery Institute, Nuffield Department of Medicine, University of OxfordOxford, UK

**Keywords:** ubiquitin, deubiquitylating enzyme, transcription, epigenetics, DNA damage response, small molecule inhibitors, multiple myeloma, cell cycle checkpoints

## Abstract

Controlling cell proliferation is one of the hallmarks of cancer. A number of critical checkpoints ascertain progression through the different stages of the cell cycle, which can be aborted when perturbed, for instance by errors in DNA replication and repair. These molecular checkpoints are regulated by a number of proteins that need to be present at the right time and quantity. The ubiquitin system has emerged as a central player controlling the fate and function of such molecules such as cyclins, oncogenes and components of the DNA repair machinery. In particular, proteases that cleave ubiquitin chains, referred to as deubiquitylating enzymes (DUBs), have attracted recent attention due to their accessibility to modulation by small molecules. In this review, we describe recent evidence of the critical role of DUBs in aspects of cell cycle checkpoint control, associated DNA repair mechanisms and regulation of transcription, representing pathways altered in cancer. Therefore, DUBs involved in these processes emerge as potentially critical targets for the treatment of not only hematological, but potentially also solid tumors.

## Introduction

Posttranslational modifications dictate the fate and function of most proteins. Chemical modifications by phosphate groups and ubiquitin, a small 76 amino acid protein, are amongst the most common ones. Targeting enzymes that modulate protein phosphorylation, such as protein kinases, has been proven to be a suitable inroad to novel anti-cancer therapeutics. In the case of the ubiquitin system, drug development efforts have been lagging behind due to the complexity of the ubiquitin conjugating and deconjugating mechanisms, and because many aspects of the fundamental biology of this pathway, in particular the topology of poly ubiquitin chains and post-translational modifications present on ubiquitin itself, are not yet fully understood (Cohen and Tcherpakov, 2010; Swatek and Komander, 2016). Despite this, the clinical approval of the proteasome inhibitors Bortezomib, Carfilzomib, and Ixazomib has boosted new drug discovery programs targeting different components of the ubiquitin system (Adams, 2002; Cohen and Tcherpakov, 2010; Ernst et al., 2013; Herndon et al., 2013; Shirley, 2016).

The ubiquitin system is involved in the regulation of almost every cellular activity through proteolytic and non-proteolytic events, including protein degradation by the 26S proteasome or through the lysosomal pathway and autophagy, protein–protein interactions, protein activity and protein localization (Herndon et al., 2013; Swatek and Komander, 2016). The covalent attachment of ubiquitin to a target protein is catalyzed by the sequential action of three enzymes: E1 activating enzyme, E2 conjugating enzyme and E3 ligase. In the final ubiquitylation step, Ub is usually transferred to an ε-NH_2_ of a lysine residue in the target protein. The addition of one or more ubiquitin monomers to another substrate-attached ubiquitin is possible and leads to formation of polymeric chains. There are different types of Ub polymers depending on the linkage and on the topology of the chain. The process is reversible and the removal of ubiquitin is catalyzed by a subclass of isopeptidases referred to as deubiquitylating enzymes or DUBs (Hershko and Ciechanover, 1998; Komander and Rape, 2012; Mevissen et al., 2013; Swatek and Komander, 2016).

## Deubiquitylating Enzymes

There are ∼90 DUBs encoded in the human genome, which are sub classified into seven different families: ubiquitin-specific proteases (USPs), ubiquitin carboxy-terminal hydrolases (UCHs), ovarian tumor domain con-taining proteases (OTUs), Machado-Joseph disease protein domain proteases (MJD), JAMM/MPN domain-associated metallopeptidases (JAMMs), the monocyte chemo-tactic protein-induced protein (MCPIP) and the motif interacting with Ub-containing novel DUB family (MINDY). Apart from the JAMMs family which has zinc metalloprotease activity, DUBs are cysteine proteases (Komander et al., 2009; Fraile et al., 2012; Kolattukudy and Niu, 2012; Abdul Rehman et al., 2016).

DUB hydrolase activity (predominantly IsoT/USP5) is required to generate free ubiquitin from its precursors because Ub is transcribed as a fusion of multiple Ub molecules or as a fusion with other proteins (Hadari et al., 1992). Keeping a steady-state level of free Ub is essential for cell viability (Wang C.H. et al., 2014). Therefore, a second process involving DUBs is the recycling of ubiquitin by preventing its degradation, which is mediated by proteasome associated DUBs USP14, UCH-L5/UCH37, and POH1, or receptor mediated endocytosis and lysosomal degradation associated DUBs USP8 and AMSH (Reyes-Turcu et al., 2009; Lee et al., 2011). More specifically, DUBs antagonize the action of ubiquitin E3 ligases that target protein substrates for degradation or by regulating E3 ligases activity and/or stability. Generally, the addition of Ub monomers or polymers to a protein can also generate non-proteolytic signals. Thus, DUBs can modulate the outcome of those signals by two main mechanisms: by removing ubiquitin polymers or monomers from proteins involved in these signaling events, but also by editing the linkage and topology of the ubiquitin chains present in the substrate (Komander et al., 2009; Bennett, 2010; Fraile et al., 2012; Komander and Rape, 2012).

Deubiquitylating enzymes activity can be tightly regulated by different means, including transcriptional changes in their gene expressions, microRNAs, post-translational modifications including phosphorylation and auto-ubiquitylation, protein interactions and by changing their subcellular localization (Komander et al., 2009; Fraile et al., 2012; Yang et al., 2012; Wijnhoven et al., 2015). Despite that the number of encoded DUBs is moderate, it is anticipated that most of them act on a discrete set of protein substrates due to restrictions in Ub chain linkage recognition as observed for OTUs (Mevissen et al., 2013) or metalloprotease DUBs (Komander et al., 2009), or the requirement of interactions with specific adaptors or scaffold proteins, as noted for JAMMs and a subset of USPs (Ventii and Wilkinson, 2008; Komander et al., 2009; Rahighi et al., 2009; Reyes-Turcu et al., 2009; Bremm et al., 2010; Mevissen et al., 2013).

As we will highlight in this review, DUBs emerge as regulators of many cellular signaling pathways critical for cell survival, proliferation, genome stability, and transcriptional control, all of which are important processes that when altered can contribute to the development of neoplasia and tumorigenesis. In particular, ubiquitylation events linked to chromatin-dependent processes appear to involve a large subset of E3 ligases and DUBs that are recognized to be prominent targets in cancer. The possibility of using small molecule inhibitors against DUBs as inroads for anti-cancer strategies are now receiving a prominent focus in pharma and academia (Nicholson et al., 2007; Sgorbissa et al., 2010; Shi and Grossman, 2010; Edelmann et al., 2011; Fraile et al., 2012; Lim and Baek, 2013; McClurg and Robson, 2015; Lim et al., 2016).

## DUBs Affecting Chromatin Function

Histone and other chromatin-associated protein modifications, together with DNA methylation, provide the cell with long-term epigenetic gene transcription regulations without affecting its DNA sequence. Deregulation of these processes is a common event in cancer development and progression (Esteller, 2007; Gronbaek et al., 2007; Segal and Widom, 2009; Simo-Riudalbas and Esteller, 2015). Most histones are post-translationally modified by the addition of different molecules including acetylation, methylation, phosphorylation, SUMOylation, and ubiquitylation. All these histone PTMs (post-translational modifications) play an important role in gene transcription regulation and chromatin remodeling, but also in the DNA damage response (DDR; Weake and Workman, 2008; Zhou et al., 2009; Zhang T. et al., 2015). Consequences of histone ubiquitylation have been extensively reviewed (Weake and Workman, 2008; Belle and Nijnik, 2014). Up to 10% of cellular histone H2A is mono-ubiquitylated on lysine 119 (H2AK119Ub), and this modification is crucial for the regulation of transcription, cell cycle progression, and DDRs (Clague et al., 2015). Poly-ubiquitylation of the same histone H2A (and its variant H2AX) on K13/15 is important for the DDR. In the case of Histone H2B, H2BK120Ub has been identified as a marker of gene activation. Other histones are also ubiquitylated, such as histone H3, which has an important role in nucleosome assembly. These histone modifications seem to be non-redundant, and whereas ubiquitylation of histone H2B is related to transcription activation and silencing, ubiquitylated H2A accumulates at repressed promoters. Not surprisingly, a number of DUBs have been identified as histone modifiers (listed in **Table 1**). Some of these DUBs present specificity for H2A, others are specific for H2B, but many of them present dual specificity toward these two histones (see **Table 1**). The high number of DUBs targeting histones suggests redundant or context-specific roles for these enzymes (Nakagawa et al., 2008; Feng et al., 2010; Cao and Yan, 2012; Lee et al., 2013; Mosbech et al., 2013; Zhang et al., 2013). The regulation of histones by DUBs has already been linked to cancer. Two good examples are USP22 and BAP1. USP22 deubiquitylates both, H2A and H2B (Zhang et al., 2008a,b; Atanassov et al., 2009; Wang and Dent, 2014). In a recent clinicopathological study in colon carcinoma samples, ubiquitylation of H2B (uH2B) was found to be decreased in colon cancers as compared to normal colon epithelium. Interestingly, high expression levels of USP22 in these tumor samples statistically correlated with reduced levels of uH2B (Wang Z. et al., 2015). USP22 has been linked to poor prognosis in cancer, making it a very attractive target in cancer research. Overexpression of this DUB has been found in colorectal cancer (Liu Y.L. et al., 2011), gastric cancer (Yang et al., 2011; He et al., 2015), liver cancer (Tang et al., 2015a,b), breast cancer (Zhang Y. et al., 2011), glioma (Liang J. et al., 2014), pancreatic cancer (He et al., 2015), non-small-cell lung cancer (Hu et al., 2012), salivary adenoid cystic carcinoma (Dai et al., 2014), human pharyngeal squamous cell carcinoma (Dou et al., 2014), and oral squamous cell carcinoma (Piao et al., 2012). BAP1 is a nuclear DUB that targets histone 2A mono-ubiquitylation on lysine 119 (H2AK119ub1; as part of the Polycomb repressor unit; Scheuermann et al., 2010) and regulates histone H3 lysine methylation and chromatin functions (Dey et al., 2012). The forkhead transcription factor FOXK2 acts a scaffold protein between BAP1 and DNA, promoting targeted epigenetic regulation by BAP1 (Ji et al., 2014; Okino et al., 2015). BAP1 has been identified as a tumor suppressor and as a potential prognostic marker for a number of cancer types. Both, germline and somatic mutations and nuclear expression loss of BAP1 have been linked to increased susceptibility and poor prognosis in malignant melanocytic proliferations (Wiesner et al., 2011; Piris et al., 2015), mesothelioma (Bott et al., 2011), basal cell carcinoma (Mochel et al., 2015), meningioma (Abdel-Rahman et al., 2011), lung cancer (Abdel-Rahman et al., 2011), bladder cancer (Nickerson et al., 2014), thymic carcinoma (Wang Y. et al., 2014), and clear cell renal cell carcinoma (Murali et al., 2013). Although the majority of the published studies suggest that the effects of BAP1 in cancer involve a disruption of the epigenetic homeostasis in these tumors (Wang Y. et al., 2014), for some researchers, it is not so clear that the antitumor effect of BAP1 is only dependent on H2A deubiquitylation (Pena-Llopis et al., 2012).

**Table 1:** Selection of deubiquitylating enzymes (DUBs) involved in modulating histone H2A/B ubiquitylation.

DUB	Histone (substrate)	Process	Selected reference
USP3	H2A, γH2AX and H2B	Cell cycle and DNA double-strand break response	Nicassio et al., 2007
USP7	H2A and H2B	Gene expression	van der Knaap et al., 2005; Luo et al., 2015
USP10	H2A (H2A.Z)	Transcriptional activation	Draker et al., 2011
USP12	H2A and H2B	*Xenopus* development	Joo et al., 2011
USP16	H2A	Cell cycle and gene expression	Joo et al., 2007; Gu et al., 2016
USP21	H2A	Transcriptional activation	Nakagawa et al., 2008
USP22	H2A and H2B	Embryonic development and telomere integrity	Zhang et al., 2008a,b; Atanassov et al., 2009; Wang and Dent, 2014
USP29	H2A and H2B	DNA double-strand break response	Mosbech et al., 2013
USP36	H2B	Unknown	Taillebourg et al., 2012
USP44	H2A and H2B	DNA double-strand break response (H2A) and stem cell differentiation (H2B)	Fuchs et al., 2012; Mosbech et al., 2013
USP46	H2A and H2B	*Xenopus* development	Joo et al., 2011
USP49	H2B	Co-transcriptional pre-mRNA processing	Zhang et al., 2013
BAP1	H2A	Gene expression	Scheuermann et al., 2010
OTUB1	Histones (unspecified)	DNA double-strand break response	Sato et al., 2012
BRCC36	H2A and γH2AX	DNA double-strand break response	Shao et al., 2009
MYSM1	H2A	Gene expression	Zhu et al., 2007; Jiang et al., 2015; Li et al., 2016

## DUBs Role in the Crosstalk Between the Different Histone PTMs

The crosstalk between different histone PTMs has been described (Zhang T. et al., 2015). JAMM/MPN, a member of the domain-associated metallopeptidases, plays an important role in gene expression regulation by coordinating acetylation of histones with deubiquitylation of histone H2A and regulating by this way the association of histone H1 with nucleosomes (Zhu et al., 2007). USP22 has been found to be associated with the Spt-Ada-Gcn5-acetyltransferase (SAGA) histone acetyltransferase (HAT) complex. In this context, USP22 deubiquitylates histone H2B and other components of the shelterin complex (Atanassov et al., 2009). USP17 regulates histone acetylation through deubiquitylation of K63-polyubiquitylated SDS3, inhibiting the histone deacetylase activity (HDAC) of SDS3 and subsequently the proliferation and anchorage-independent growth of tumor cells (Ramakrishna et al., 2011, 2012). A recent study describes that USP7 interacts with and deubiquitylates Tip60, an acetyltransferase targeting histones, resulting in its stabilization (Dar et al., 2013). As mentioned above, BAP1 forms together with ASXL1 the PR-DUB complex that removes ubiquitin from H2AK119Ub. In a recent study, LaFave et al. (2015) found that BAP1 deletion in mice increased the levels of tri-methylated histone H3 (H3K27me3) and reduced mono-methylation of the histone H4 (H4K20me1). A member of the OTU family of DUBs called TRABID (also ZRANB1) was found to be an innate immunological regulator of inflammatory T cell responses. TRABID regulates histone methylation (H3K9me2, H3K9me3, and H3K4me3) at the promoter of IL-12 by deubiquitylating and stabilizing the histone demethylase JMJD2D (Jin et al., 2016). In the same study, ectopic expression of TRABID reduced K29, and to a lesser extent K11 ubiquitylation of JMJD2D. This data is consistent with previous published studies describing specificity of this DUB toward K29-linked ubiquitin chains in *in vitro* assays (Virdee et al., 2010; Licchesi et al., 2012). The protein TIP5 is part of the nucleolar remodeling complex (NoRC) that modulates the silencing of a fraction of rDNA by recruiting histone and DNA methyltransferases. TIP5 is deubiquitylated and stabilized by USP21, resulting in an increase of H3K4me3 and rDNA promoter methylation (Khan et al., 2015). It had been previously described that H2A ubiquitylation controls the di- and tri-methylation of H3K4. In the same study, the authors describe the indirect effects of USP21 on H3K4m2, and H3K4m3 modifications through its H2A histone deubiquitylating activity (Nakagawa et al., 2008). An interesting study has recently linked the roles of the DUB USP7 on epigenetic regulation, cell cycle, and DNA repair (Wang Q. et al., 2016). Upon DNA damage, USP7 interacts, deubiquitylates and stabilizes the histone demethylase PHF8, inducing the specific expression of a subset of genes, including the cell cycle regulator cyclin A2. In the same article, USP7, PHF8, and cyclin A2 were found to be overexpressed in breast carcinomas, correlating with the histological grade of disease. USP24 was also found to target histones by controlling the levels of the histone-lysine *N*-methyltransferase Suv39h1, resulting in a modulation of the H3K9me levels. Single-nucleotide polymorphisms (SNPs) of USP24 were found in lung cancer. The variants 930C/T and 7656T/C were increased in tumor samples and were found to induce USP24 expression by stabilizing RNA. (Wang Y.C. et al., 2015). LSD1 (lysine-specific demethylase 1) removes methyl groups from H3K4 and also from H3K9. LSD1 has been found to be upregulated in many tumors and its protein levels regulated by the ubiquitin proteasome system. Using a library of siRNA against all human DUBs, USP28 was found as the DUB involved in the stabilization of LSD1. USP28 interacts and deubiquitylates LSD1, and the expression levels of the two proteins correlate well in tumor cell lines and tumor samples (Wu et al., 2013). In conclusion, all these examples are starting to point toward an emerging and important role of the DUBs in the regulation of gene expression by epigenetic events in cancer.

## DUBs and DNA Methylation

Only one DUB, USP7, has been described to have a role in the DNA methylation process. USP7 regulates the inheritance of DNA methylation patterns through control of the abundance of Dnmt1, the DNA methyltransferase responsible for this epigenetic mark (Bronner, 2011; Qin et al., 2011). USP7 is part of a protein complex with Dnmt1, the histone acetyl transferase Tip60 and the ubiquitin ligase Uhrf1. USP7 and Urf1 tightly regulate the abundance of Dnmt1 in order to control DNA methylation inheritance and replication of methylation patterns. Supporting the role of ubiquitylation in the maintenance of DNA methylation, Nishiyama et al. (2013) have discovered that the ubiquitylation of histone H3 is necessary to keep the DNA methylation mark by Dnmt1.

## DUBs and DNA Damage

**FIGURE 1:**
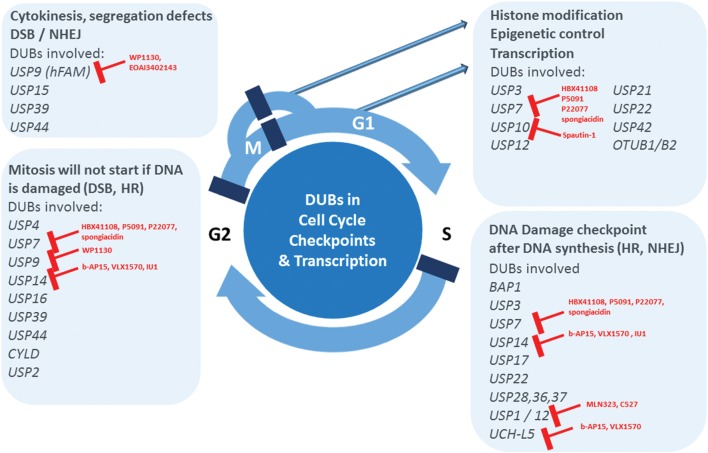
**Deubiquitylating enzymes (DUBs) emerge as major pharmacological targets.** Uncontrolled cell proliferation is one of the hallmarks of cancer. Recent literature evidence establishes the critical role of DUBs in aspects of cell cycle checkpoint control, associated DNA repair mechanisms and regulation of transcription, representing pathways altered in cancer. Therefore, DUBs involved in these processes emerge as potentially critical targets for the treatment of not only hematological, but potentially also solid tumors. Small molecule inhibitors available for a subset of DUBs are indicated in red.

In order to achieve the mutational status that leads to malignancy, tumor cells often deregulate the DDR and the genome maintenance systems (Hanahan and Weinberg, 2011). Under normal physiological conditions, cells have sensor proteins that check for damage in their genome, and once these proteins detect the lesion, repair enzymes are recruited to the damage site to promote repair. Small lesions are repaired by base excision repair (BER), nucleotide excision repair (NER), mismatch repair (MMR) and the Fanconi anemia (FA) pathways. More harmful damages such as double-stranded breaks (DSBs) can be repaired by non-homologous end joining (NHEJ) or through homologous recombination (HR). All the DDR pathways are tightly regulated by PTMs, which includes ubiquitylation/deubiquitylation events. Therefore, DUBs are involved in multiple DDR checkpoints in addition to their capability to modulate histone modifications (**Table 2** and **Figure 1**; Bennett and Harper, 2008; Jacq et al., 2013; Liu et al., 2016; Nie and Boddy, 2016). FA complementation group D2 protein (FANCD2) and proliferating cell nuclear (PCNA), when mono-ubiquitylated, induce the DDR. USP1 targets FANCD2, FANC1, and PCNA for deubiquitylation. FANCD2 and FANC1 are implicated in the FA pathway and PCNA in the translesion synthesis process (Nijman et al., 2005; Oestergaard et al., 2007; Hendel et al., 2011; Villamil et al., 2013; Kim and Kim, 2016). USP1 mutations and deregulated expression levels have been reported in different tumors (Garcia-Santisteban et al., 2013). The Chk2-p53-PUMA pathway is another regulator of the DDR generated by double-strand breaks. USP28 was found to stabilize two components of this pathway upon DNA damage, Chk2 and 53BP1 (Zhang et al., 2006). The proteasome-associated DUB Rpn11/POH1 has been described as an important regulator of ubiquitin conjugates generated after DNA damage, thereby representing an important component of the double-strand break response (Butler et al., 2012). BRIT1 is an early DDR factor that is recruited upon DSBs by phosphorylated H2AX histone (γ-H2AX), and it contributes to the final repair process by inducing chromatin relaxation. BRIT1 is deubiquitylated and stabilized by USP8 with the help of the scaffold protein BRUCE, tightly regulating the action of BRIT1 at damaged sites (Ge et al., 2015). USP4 has been found to be important for DSB repair by promoting homologous recombination. USP4 is auto-deubiquitylated on lysine, but potentially also on cysteine residues, and its deubiquitylation is an important step to permit interaction with the DNA-end resection factor CtIP and MRE11-RAD50-NBS1 (MRN) complex, thereby recruiting CtIP to the damaged sites (Wijnhoven et al., 2015). USP10 was recently identified as a partner of MSH2 by mass spectrometry-based interactome studies. MSH2 is an important factor for the mismatch repair pathway and for the resistance to DNA-damaging agents. USP10 stabilizes MSH2, and knockdown of USP10 in lung cancer cells reduces the sensitivity of these cells to DNA damaging agents. A well-known mechanism of DDR involves histone H2AX, which is phosphorylated and accumulated at damaged sites. Then, the ubiquitin ligases RNF168 and RNF8 ubiquitylate γ-H2AX, thereby inducing the accumulation of repair factors. USP7 has been identified as a regulator of γ-H2AX and H2A ubiquitylation by modulating the stability of the E3 ubiquitin ligases RNF168, RINGB1, and BMI1 (Zhu et al., 2015). USP7 is a regulator of different pathways of the DDR, and it is also a modulator of the ATR-Chk1 pathway since it controls the levels of two main components of this response, Chk1 (an essential checkpoint kinase in the DDR) and Claspin (an important component of the ATR-Chk1 axis) (Faustrup et al., 2009; Alonso-de Vega et al., 2014). Other DUBs have also been identified to stabilize Claspin and, therefore, to modulate the ATR-Chk1 pathway, such as is the case for USP9X (McGarry et al., 2016), USP29 (Martin et al., 2015), and USP20 (Yuan et al., 2014; Zhu et al., 2014). In an interesting study, Nishi et al., identified UCH-L5 as the only DUB from a library of 90 DUBs that were able to promote changes in the DDR during three different assays: recruitment at the damaged sites, DDR signaling modulation and DSB repair. The authors found that UCH-L5 interacted with and stabilized NFRKB. NFRKB is a component of the INO80 complex that promotes HR and DNA-end resection (Nishi et al., 2014).

**Table 2:** Deubiquitylating enzymes associated with DNA damage responses (DDR).

DUB	Non-histone substrate	DDR pathway	Selected reference
USP1	FANCD2, FANCI and PCNA	Fanconi anemia, post-replication repair (PRR) and translesion DNA synthesis (TLS)	Nijman et al., 2005; Oestergaard et al., 2007; Hendel et al., 2011; Villamil et al., 2013; Kim and Kim, 2016
USP2a	Mdm2	p53	Stevenson et al., 2007
USP4	Auto-deubiquitylation and ARF-BP1	DSB-response (HR), p53	Zhang X. et al., 2011; Wijnhoven et al., 2015
USP5	p53	p53	Dayal et al., 2009
USP7	Mdm2, p53, Claspin, Chk1, Ring1b, Bmi1 and RNF168	p53, ATR-Chk1 and γ-H2AX (DSBs and SSBs)	Brooks et al., 2007; Faustrup et al., 2009; Brooks and Gu, 2011; Alonso-de Vega et al., 2014; Qian et al., 2015; Zhu et al., 2015
USP8	BRIT1	BRIT1–SWI–SNF DSB-response	Ge et al., 2015
USP9X	Claspin	ATR-Chk1	McGarry et al., 2016
USP10	p53 and MSH2	ATM-p53 and mismatch repair (MMR)	Yuan et al., 2010; Zhang et al., 2016
USP11	p53	DDR to etoposide	Ke et al., 2014
USP20	Claspin	ATR-Chk1	Yuan et al., 2014; Zhu et al., 2014
USP24	p53 and DDB2	p53-PUMA	Zhang L. et al., 2012, 2015
USP28	Chk2 and 53BP1	Chk2-p53-PUMA	Zhang et al., 2006
USP29	p53 and Claspin	p53 and ATR-Chk1	Liu et al., 2011a; Martin et al., 2015
UCH-L5	NFRKB	DSB-response (HR)	Nishi et al., 2014
OTUB1	p53	p53	Sun et al., 2012
OTUD5	p53 and PDCD5	p53	Luo et al., 2013; Park et al., 2015
Rpn11	Ubiquitin conjugates generated by DNA damage	DNA double-strand break response	Butler et al., 2012


## Regulation of p53, c-Myc and Other Oncogenes By DUBs

The tumor suppressor p53 is a transcription factor able to control important cellular pathways including DDR, cell cycle, apoptosis, angiogenesis, and senescence. It is called “the guardian of the genome” because of its ability to prevent genome mutation and tumor onset and progression (Nag et al., 2013). p53 levels and subcellular localization are mainly regulated by ubiquitylation (Brooks and Gu, 2011). A number of DUBs can modulate p53 signals: USP7 deubiquitylates both p53 and MDM2, one of the ubiquitin ligases that ubiquitylates p53, thereby stabilizing both proteins (Brooks et al., 2007; Brooks and Gu, 2011). Upon DNA damage, USP10 deubiquitylates and activates p53. The same study shows that USP10 suppresses tumor cell growth in cells expressing wild-type p53 (Yuan et al., 2010). USP2a associates with and deubiquitylates MDM2, but not p53, and promotes MDM2-dependent p53 degradation (Stevenson et al., 2007). Overexpression of this DUB was found in different tumors such as glioma (Boustani et al., 2016), bladder cancer (Jeong et al., 2015), prostate cancer (Nelson et al., 2012), and oral squamous carcinoma (da Silva et al., 2009). USP4 interacts with and deubiquitylates another E3 ubiquitin ligase for p53, ARF-BP1/Mule/HUWE, leading to the stabilization of ARF-BP1 and subsequent reduction of p53 levels. The same authors found that USP4 is overexpressed in several types of human cancer, and they suggest that USP4 could be a potential oncogene (Zhang X. et al., 2011; Xing et al., 2016). In response to oxidative stress, USP29 binds to and stabilizes p53. Accumulated p53 quickly induces apoptosis under these conditions (Liu et al., 2011a). OTUB1 is a DUB with preference for substrates with poly ubiquitin K48 linked chains (Mevissen et al., 2013; Altun et al., 2015) that was reported to suppress MDM2-mediated p53 ubiquitylation (Sun et al., 2012). Interestingly, the mechanism by which OTUB1 controls p53 ubiquitylation is independent of its DUB activity. OTUB1 blocks p53 ubiquitylation by MDM2 by interacting with and inhibiting UbcH5, and E2 conjugating enzyme for MDM2. Overexpression of OTUB1 in cells drastically stabilizes and activates p53, leading to apoptosis and to a marked inhibition of cell proliferation in a p53-dependent manner (Sun et al., 2012). OTUB1 is also involved in the inhibition of another E2 enzyme, UBC13 (Sato et al., 2012). Since UBC13 is the only E2 involved in the conjugation of K63 Ub chains, this makes OTUB1 an interesting regulator of both, K63 and K48 polyubiquitin chains signals. OTUB1 is also an activator of a very important oncogene in cancer, RAS. Remarkably, OTUB1 deubiquitylates mono- and di-ubiquitylated RAS, independently of its activation status, resulting in the translocation of the RAS protein to the plasma membrane where it is normally activated (Baietti et al., 2016). OTUB1 expression levels are related with poor prognosis and metastasis in colorectal cancer (Zhou et al., 2014), ovarian cancer (Wang Y. et al., 2016), non-small-cell lung carcinomas (Baietti et al., 2016), and it has been linked to resistance to chemotherapy in breast cancer bearing patients (Karunarathna et al., 2016) and prostate cancer cell invasion (Iglesias-Gato et al., 2015). Knock-down of USP5, the only DUB with specificity for unanchored poly ubiquitin, stabilizes p53 as well. The authors propose a model in which p53 is selectively stabilized because the unanchored poly ubiquitin that accumulates after USP5 knockdown is able to compete with ubiquitylated p53 for proteasomal proteolysis (Dayal et al., 2009). Another DUB, OTUD5, interacts and deubiquitylates p53 in response to DNA damage stress (Luo et al., 2013). PDCD5 is an additional factor accumulated upon DNA damage and regulates the p53 pathway. In a two yeast two-hybrid study to identify partners of PDCD5 in the presence of genotoxic stress, OTUD5 was found to interact with and stabilize PDCD, thereby unveiling a dual role for this enzyme in the regulation of the p53 signals (Park et al., 2015). In another two-hybrid study, USP24 was found to interact with and stabilize DDB2 (UV damage binding protein; Zhang L. et al., 2012). Posteriori, the same authors identified an upregulation of USP24 in a number of tumor cell lines. They found also that USP24 deubiquitylates p53, activating the PUMA pathway, a regulator of DNA-damage-induced apoptosis (Zhang L. et al., 2015). Similarly, it was found that USP11 deubiquitylates p53 in response to genotoxicity induced by etoposide (Ke et al., 2014). Other DUBs are linked to the turn-over of important tumor suppressors and oncogenes. For instance, oncogenic transformation by the stabilization of the dual specificity (Tyr/Thr) phosphatase Cdc25A appears to be controlled by Dub3/USP17 (Pereg et al., 2010). USP17 knockdown does lead to a cell cycle arrest in G1/S and G2/M, and high levels of USP17 have been observed in lung, colon, esophagus, and cervix tumor biopsies, underpinning its role in cell cycle control (McFarlane et al., 2010). USP7 is abundantly expressed in many cell types and, as mentioned above, it was shown to deubiquitylate MDM2, thereby modulating p53 stability, but it also has other cellular substrates including FOXO4, Claspin and FOXO3 (Nicholson and Suresh Kumar, 2011). Knockdown of USP7 leads to cell cycle arrest in G1 or G2 (Khoronenkova et al., 2012), which is underpinning its role in controlling several aspects of cell division. The JAMM-domain containing DUB BRCC36 stimulates activity of BRCA1, leading to G2/M checkpoint arrest/control (Mallery et al., 2002). In addition to its role in the p53 pathway, USP28 was also shown to stabilize the oncogene c-MYC after DNA damage. The same authors and others found high expression levels of this DUB in colon, lung, glioma, bladder, and breast carcinomas (Popov et al., 2007a,b; Diefenbacher et al., 2014, 2015; Guo et al., 2014; Wang Z. et al., 2016). USP36 and USP37 also control the stability of c-Myc and thereby affect c-Myc oncogene driven cellular proliferation (Zhang et al., 2006; Pan et al., 2015; Sun et al., 2015).

## DUBs Affecting Cell Cycle Regulators

The accumulation and turnover of proteins that regulate the cell cycle such as cyclins, CDKs, and checkpoint signaling molecules is highly orchestrated and controlled to ensure the timely progression through the cell cycle. Inappropriate expression of one or more of these proteins is a common feature of virtually all human tumors. A large number of studies have underscored the importance of E3 ubiquitin ligases and the role they play in regulating cell cycle components [reviewed in Li and Jin (2012)]. However, DUBs that counterbalance E3 ligase activity may also be critical in cell cycle progression (Song and Rape, 2008; Fraile et al., 2012). Indeed, DUB function is frequently miss- regulated in cancer, and our knowledge concerning DUB expression and activity during the different phases of the cell cycle is expanding (for a specific review, see Lim et al., 2016). The regulation of chromatin structure and transcription is one of the key mechanisms by which DUBs exert cell cycle control. DUBs involved in DNA damage checkpoints are exerting effects on cell cycle progression, and these include USP1, USP3, USP7, USP10, USP11, USP16, USP21, USP22, USP28, BRCC36, MYSM1, and BAP1 (Jacq et al., 2013; see also section above). Another key cell cycle checkpoint is the one controlling the correct mitotic spindle assembly, and DUBs such as USP44, CYLD, and USP15 were reported to modulate this process. USP44 acts as a tumor suppressor by preventing chromosome segregation errors (Holland and Cleveland, 2012) via deubiquitylation of the anaphase promoting complex (APC) coactivator Cdc20 (Stegmeier et al., 2007a), and USP44 deletion leads to spontaneous tumor formation, preferentially in the lungs (Zhang Y. et al., 2012). USP15 stabilizes newly synthesized REST and rescues its expression at mitotic exit (Faronato et al., 2013). CYLD targets Plk1 and contributes to regulating mitotic entry (Stegmeier et al., 2007b). USP3 modifies chromatin and is required for S-phase progression (Nicassio et al., 2007). USP2a, as mentioned above, has been linked to different types of cancer. In bladder cancer cells, USP2a was found to deubiquitylate and to stabilize the cell cycle regulator, Cyclin A1, controlling proliferation of these cells (Kim et al., 2012). Taken together, ∼15 (out of ∼90) DUBs have been directly linked to molecular processes of the cell cycle (**Figure 1**).

## Will DUB Inhibition Work in Cancer?

Generally, DUB function is linked to most cellular processes, but in particular appears to cluster around three major pathways that are commonly deregulated in tumorigenesis. These include transcriptional and epigenetic control of gene expression, DDR pathways and cell cycle checkpoint control (**Figure 1**). These processes are functionally interconnected, for instance DNA repair mechanisms that are part of cell cycle control checkpoints in the transitions from G2 to S and M to cytokinesis. The associated subset of DUBs are therefore attractive drug candidates, although so far small molecule inhibitors for only a few of them have been reported many of them are subject to intense screening activities (for a comprehensive review, see Kemp, 2016) including natural compounds (Tsukamoto, 2016). The most relevant DUBs related to these pathways with existing chemical matter are UCH-L1, USP1, USP7, USP9, USP14, and UCH-37. Inhibitors of the USP1/UAF complex have been reported, such as ML323, developed based on a N-Benzyl-2-phenylpyrimidin scaffold (Liang Q. et al., 2014), and C527 (SJB3-019A; Mistry et al., 2013). Interestingly, ML323 was found to sensitize the non-small lung cancer cell line H596 to cisplatin (Liang Q. et al., 2014). Most efforts so far have been focused on USP7 (HAUSP), because of its effect on the MDM2-p53 axis. Reported inhibitors include Hybrigenix HBX41108/HBX19818, all based on quinazoline core structures (Colland et al., 2009; Reverdy et al., 2012), Progenra P22077/P5091 developed from a phenyl-thio-2-thienyl building block (Altun et al., 2011; Chauhan et al., 2012), but also natural compounds such as spongiacidin (Yamaguchi et al., 2013). Most promisingly, the USP7 inhibitor P5091 was shown to be able to overcome Bortezomib resistance in Multiple Myeloma cells (Chauhan et al., 2012). However, this chemical scaffold has limited pharmacodynamics properties. A different small molecule, WP1130 and its improved derivative EOAI3402143 based on second-generation tyrphostin derivatives [initially identified as Janus-activated kinase (JAK)-signal transducer], appear to inhibit USP9X and USP24 and consequently increased Myeloma tumor cell apoptosis *in vitro* and *in vivo* (Peterson et al., 2015). USP9X affects chromosome alignment and segregation via ubiquitylation of survivin (Vong et al., 2005), but has potentially other roles including cell sensitization by affecting the stability of MCL-1, BCR-ABL, and ITCH (Schwickart et al., 2010; Kushwaha et al., 2015). Spautin-1, a quinazolinamine derivative, was characterized as a USP10 inhibitor that to some extent also targets USP13 (Liu et al., 2011b). The resemblance of the quinazoline chemical core element to USP7 inhibitors may offer an opportunity to use this scaffold to further develop inhibitors specific for other USPs.

## Clinical Perspective for DUB Inhibitors

The USP14 inhibitor IU1 and dual inhibitors of USP14/UCHL5 (proteasomal DUBs) such as b-AP15 (D’Arcy et al., 2011), a *bis*[(4-nitrophenyl)methylene]-piperidinone derivative or its more recently developed analog VLX1570 (Wang X. et al., 2015), show potentially promising effects that could be potentially translated into the clinic. IU1 appears to accelerate degradation of protein aggregates (Lee et al., 2010), and b-AP15 also overcomes Bortezomib resistance in Multiple Myeloma cells (Tian et al., 2014). As a consequence, VLX1570 has now been cleared to enter Phase I/II for the treatment against Multiple Myeloma patients for whom every other drug combination failed (Taylor, 2016). This represents the first DUB inhibitor reaching the clinical phase, and it is expected that within the next one to two years, a number of other DUB inhibitor candidates will follow also for the potential treatment of solid tumors. For instance, p53 wildtype expressing tumors (e.g., certain colon cancers) may be suitable for USP7/10 inhibitor based treatment strategies. c-Myc-dependent tumors (adenocarcinomas and non-small lung, breast, and colon cancer) could potentially respond to USP28, USP36, USP37, all DUBs affecting c-Myc protein turnover. One third of human cancers present mutations in the oncogene RAS or in components of its effector pathways (Matallanas and Crespo, 2010). The recent discovery describing how OTUB1 control RAS activity (Baietti et al., 2016) may lead to the development of OTUB1 inhibitors targeting RAS mutation based cancers such as pancreatic cancer, colon and melanoma. Clearly, DUBs have now reached center stage as cancer targets, and novel inhibitors for this enzyme class will provide the framework for more effective single agent or combination therapies to better treat hematological and solid tumors.

## Author Contributions

BK derived the concept and structure of the manuscript and **Figure 1**, and he wrote parts of the review. AP-F wrote parts of the review, prepared the tables and contributed to proof-reading.

## Conflict of Interest Statement

AP and BK are both associated with Cancer Research Technologies and Forma Therapeutics.
